# Supporting patient decision-making in non-invasive prenatal testing: a comparative study of professional values and practices in England and France

**DOI:** 10.1186/s12910-024-01032-0

**Published:** 2024-03-21

**Authors:** Hilary Bowman-Smart, Adeline Perrot, Ruth Horn

**Affiliations:** 1 Ethox Centre, University of Oxford, Oxford, UK; 2 Monash Bioethics Centre, Monash University, Melbourne, Australia; 3 Biomedical Ethics Research Group, Murdoch Children’s Research Institute, Melbourne, Australia; 4 Australian Centre for Precision Health, Clinical and Health Sciences, University of South Australia, Adelaide, Australia; 5 Institute of Ethics and History of Health in Society, University of Augsburg, Augsburg, Germany

**Keywords:** Non-invasive prenatal testing, Supporting reproductive decision-making, Values, Practices, Policy, Comparative analysis, France, England

## Abstract

**Background:**

Non-invasive prenatal testing (NIPT), which can screen for aneuploidies such as trisomy 21, is being implemented in several public healthcare systems across Europe. Comprehensive communication and information have been highlighted in the literature as important elements in supporting women’s reproductive decision-making and addressing relevant ethical concerns such as routinisation. Countries such as England and France are adopting broadly similar implementation models, offering NIPT for pregnancies with high aneuploidy probability. However, we do not have a deeper understanding of how professionals’ counselling values and practices may differ between these contexts.

**Methods:**

In this paper, we explore how professionals in England and France support patient decision-making in the provision of NIPT and critically compare professional practices and values. We draw on data from semi-structured interviews with healthcare professionals.

**Results:**

Both English and French professionals emphasised values relating to patient choice and consent. However, understandings and application of these values into the practice of NIPT provision differed. English interviewees placed a stronger emphasis on interpreting and describing the process of counselling patients and clinical care through a “principle” lens. Their focus was on non-directiveness, standardisation, and the healthcare professional as “decision-facilitator” for patients. French interviewees described their approach through a “procedural” lens. Their focus was on formal consent, information, and the healthcare professional as “information-giver”. Both English and French professionals indicated that insufficient resources were a key barrier in effectively translating their values into practice.

**Conclusion:**

Our findings illustrate that supporting patient choice in the provision of NIPT may be held as an important value in common on a surface level, but can be understood and translated into practice in different ways. Our findings can guide further research and beneficially inform practice and policy around NIPT provision.

**Supplementary Information:**

The online version contains supplementary material available at 10.1186/s12910-024-01032-0.

## Background

Non-invasive prenatal testing (NIPT) has transformed clinical practice in the prenatal care setting over the past decade. In this paper, we explore key values regarding information provision and patient choice held by professionals involved with the implementation of NIPT, and critically compare how these influence practice across two different public healthcare systems (England and France).

NIPT is based on a maternal blood test and has a higher performance for common aneuploidies such as trisomy 21 (T21, Down syndrome) than some other screening methods for fetal conditions (e.g. combined first trimester screening). With higher test performance, uptake of NIPT is associated with a reduction in rates of diagnostic procedures such as amniocentesis [1]. In the future, the scope of NIPT may expand to include detailed analysis of the fetal genome [2].

NIPT has been rapidly and widely adopted to varying degrees across the world, in both public and private healthcare systems [3]. It has recently been introduced into several public healthcare systems across Europe, including Belgium, the Netherlands, France, Germany, and the United Kingdom (UK) [3, 4]. There are differing models for implementation, raising various ethical and practical challenges for healthcare professionals. For example, Belgium and the Netherlands have opted for variations of a first-tier model, where NIPT is partially or fully subsidised for all pregnancies [5, 6]. Other countries, such as France and England, have used a contingent model where NIPT is offered to pregnancies with a previously established higher probability of fetal aneuploidy. Germany has taken a unique approach of offering NIPT on a case-by-case basis [7]. The scope of the test also varies. In England, NHS Screening recommends that NHS Trusts only provide NIPT for the three most common trisomies (T21, as well as trisomy 18 (T18, Edwards’ syndrome) and trisomy 13 (T13, Patau’s syndrome) [8]. In France, however, many laboratories have begun to also offer genome-wide screening [9]. NIPT is also available in the private sector where there may be different options that the patient can pay out of pocket for, including tests with a wider scope (e.g. including fetal chromosomal sex).

The increasing availability of NIPT in public healthcare systems places us at the beginning of a new era of prenatal screening, raising questions about how women and prospective parents can best be supported in making reproductive decisions. Maintaining reproductive autonomy in clinical care is widely considered critical, which includes supporting informed choice about prenatal testing and subsequent decision-making related to the pregnancy [10]. However, in practice it is not always clear to professionals how to most effectively do so. As part of ensuring appropriate and ethical implementation of NIPT, it is important to understand professionals’ perspectives and values regarding the process of test provision, and how women’s decisions ought to be supported in the clinic. This includes healthcare professionals and other key stakeholders such as policy-makers, scientists, and community representatives. Beyond establishing how professionals view the test offer and communication, and associated ethical and practical challenges, it is also necessary to critically compare these perspectives between different socio-cultural contexts and healthcare systems. This will then allow us to identify possible strengths and weaknesses of differing implementation approaches, which can inform policy and clinical practice.

The data we present here are an exploration of the key values and practices of English and French professionals involved in the offer of NIPT in clinical care. The focus here is on professionals’ views on informing, communicating and counselling about NIPT both before the test, as well as after test results have been returned. The comparison of England and France is particularly useful as both have contingent models that are broadly similar but differ in some key aspects. They have differing probability cut-offs – in England, eligibility for NIPT is set at a 1:150 probability of fetal aneuploidy, whereas in France it is offered more broadly, at a 1:1000 probability [4]. Policy decisions relating to probability cut-offs were driven by a range of factors [11]. In England, cost-effective analyses focused on reducing the number of invasive diagnostic procedures through the higher positive predictive value of NIPT, while keeping overall costs close to those of the previous screening programme. In France, however, the focus was on increasing the detection rate of fetal T21, which played a role in the allocation of additional resources and the broader probability cut-off.

The present data are a subset of results from a wider comparative study assessing the ethical, policy and social issues associated with the implementation of NIPT in England, France, and Germany. This wider study involves ethical and philosophical analysis, as well as an empirical component of a qualitative interview study with healthcare professionals, patients, and other key stakeholders. The results in this paper can provide an in-depth view of some of the key differences, challenges and implications of different key values and practices in informing, communicating and counselling about NIPT in England and France.

## Methods

We conducted a semi-structured interview study to gain insight into the perspectives, values and practices of professionals, and the ethical issues they identify with regards to informing, communicating and counselling about NIPT in England and France.

### Sampling and recruitment

In order to obtain a range of different views from interviewees across professions involved in NIPT, and across geographic regions, we engaged in purposive snowball sampling. Our initial recruitment methods involved drawing on our pre-existing professional networks within prenatal genetics. We approached individual professionals asking them to forward our invitation email including a participant information sheet to their colleagues and/or professional networks. Interested professionals then contacted us via email to arrange a date for an interview. After each interview, we asked the interviewee if they could further share our invitation email with their networks. Due to our strategy, we do not know how many professionals received our invitation. We pursued this recruitment strategy until saturation was reached and no new themes emerged.

The inclusion criteria were any professionals who have been involved with, have experience with, or have other relevant knowledge regarding the provision of NIPT at various stages of testing, including post-test counselling and return of results, in England or France. Interviewees were from a range of professions. In France we recruited 17 professionals for semi-structured interviews: 5 obstetrician-gynaecologists, 5 clinical geneticists, 5 midwives, and 2 laboratory/medical biologists. In England, we recruited 27 professionals for interviews: 5 geneticists, 6 genetic counsellors, 2 obstetrician-gynaecologists, 6 midwives, 1 nurse, 2 medical biologists, 5 policy-makers/patient advocates, and 1 screening co-ordinator. There were fewer French interviewees (e.g. none with French genetic counsellors) due to the differing involvement of professionals in the provision of NIPT between the French and English contexts; furthermore, at the time of the study, policy-makers in France were in the process of revising existing policy and therefore chose not to participate. We would like to recognise that at the time of data collection some of the interviewees were not themselves practicing in England or France (1 was from Wales, 1 from Scotland, 1 from Belgium), but had previous experience and/or expertise relevant to these contexts. As the research focused specifically on the English and French provision and implementation of NIPT, interviewees were recruited based on their involvement with either of these two contexts. For the purposes of this paper, we are thus describing them as “French” or “English” interviewees. The recruitment was undertaken by AP and RH (PI).

### Data collection

Prior to the interviews, interviewees were provided with a participant information sheet detailing the purpose of the study, the funding body, and the institutional affiliation and role of the researchers. On the day of the interview, consent was obtained to conduct, record, and transcribe the interviews; to use anonymised quotes in scientific publications; to store de-identified transcripts; and to deposit these in the UK Data Archive. Consent was obtained verbally from interviewees. A copy of the consent form signed by the interviewer was then emailed to the interviewee for their records.

The interviews were conducted online via Microsoft Teams in English (by AP and RH) or in French (by AP). The interviews were of approximately 45 minutes duration each. The interviews were digitally recorded and transcribed verbatim. Interviews were conducted between June 2021 and February 2022.

The interview guides for healthcare professionals and policy-makers covered a range of topics including experiences with the implementation and/or provision of NIPT; attitudes and opinions towards NIPT; views about advantages and disadvantages associated with NIPT; views about decision-making and the counselling process; and perceptions on the future possibilities of NIPT (see supplementary files).

Once data were collected, the participant’s name was replaced by a unique participant number (pseudonymisation via a linkage list). The password protected list of participants names and contact details is accessible only to AP and RH (PI) and will be kept for at least three years after publication or public dissemination, and then will be destroyed.

#### Ethics approvals

have been obtained from the University of Oxford Central Research Ethics Committee (R64800/RE001) in the United Kingdom, and the Inserm Ethics Evaluation Committee (Inserm Ethics Evaluation Committee (CEEI)/Institutional Review Board (IRB): Avis n°21–82), France.

Data are available from the UK Data Archive (DOI 10.5255/UKDA-SN-856508) for researchers who meet the criteria for access to confidential data.

### Data analysis

Following a thematic analysis approach, the interviews were coded first separately and then cross-coded by HBS, AP and RH [12, 13]. All three researchers are trained and experienced qualitative researchers. The collaborative coding involved regular meetings between the three researchers to discuss and review the construction of the meaning of the codes, in general and with regard to their cultural and linguistic translatability, and their applicability to each country’s dataset. By doing so, a master codebook was developed and applied to the interview data using NVivo software [12].

We wrote memos to develop the analysis and to generate and develop themes through constant comparison with the data. The process of comparison involved the identification not just of similarities and differences in the content of what was discussed, but also examining more deeply how interviewees articulated and framed their experiences and perspectives. This required the researchers to build on their own positionality (particularly in terms of cultural, social and linguistic background) to more meaningfully interpret the data within the context it was drawn from, and thus identify key points of comparison between the French and English data [14]. Data were then de-identified in order to protect the privacy of interviewees while retaining context and content as much as possible [15].

## Results

In this paper, we present our findings related to the key values and practices that professionals emphasised in the context of NIPT communication, counselling and provision in the English and French public health systems, and how they described and interpreted them.

Quotes from interviewees are accompanied by a participant code, with English interviewees identified as “ENG” and French interviewees as “FR”. Each quote is also accompanied by a brief description of the interviewee’s primary profession or role. As several interviewees had multiple roles or diverse professional backgrounds, we have categorised them by their self-described primary profession or role at the time of interview.

### The value and framing of “choice” and “consent”

In both the English and French contexts, interviewees emphasised the importance of a constellation of concepts relating to choice, informed consent, autonomy, decision-making, and freedom from coercion or pressure. However, how English and French interviewees described their understanding and conceptualisation of these values diverged.

Relative to English interviewees, French interviewees described choice and consent in a more procedural or legalistic way. They emphasised the importance of communicating directly to patients that NIPT and other prenatal tests are “not mandatory” and that they have the freedom to choose. They referred to existing regulation, such as the French Public Health Code.

*“…at the very beginning, (when) screening for markers…I tell them that it’s not mandatory.”* (FR-006, midwife).

*“Apart from the scientific information, of course, I make sure they know they have the choice to take up screening or not.”* (FR-007, medical biologist).

French interviewees also emphasised the importance of obtaining written consent through a formalised process as proof of free choice, which includes the option of withdrawing consent at any time. In France, it is a legal requirement that pregnant women must sign consent forms before undergoing prenatal genetic testing. Quality information must be provided to patients before they can give consent.

*“…once we have given [the women] all the information…there is a consent form with the referral and the patient must read and sign it.”* (FR-014, midwife).

*“I think that, like many things in genetics, there are already very solid bases in terms of limiting any malpractice because there is consent [and]…information [provided] beforehand.”* (FR-005, medical biologist).

In contrast, English professionals focused on “non-directiveness” as a key principle for providing a prenatal test and facilitating choice. They placed a strong emphasis on the idea that prenatal screening is a personal decision.

*“I do need to make clear as well that the central ethos of genetic counselling is non-directiveness. So, we don’t have an agenda. We would never tell a patient to have a termination or not have a termination and we never try to steer a couple into any course of action.”* (ENG-019, genetic counsellor).

English interviewees also linked “choice” with broader ethical principles and concepts such as “autonomy”. They discussed their understanding of these principles and emphasised the role of the healthcare professional in facilitating women to make decisions in line with their own personal values and circumstances.

*“At the end of the day, for me, it’s about choice and it’s about supporting people to make choices …and it’s something I say to my patients all the time: ‘I’m not here to decide for you…I’m here to support you in making a decision that you can live with and that you think is right at this moment in time.’”* (ENG-008, genetic counsellor).

*“…you know, autonomy can be, depending on what part of the world you’re from or what your beliefs are, autonomy can be different things…we’ve been thinking more about relational autonomy. So enabling people to make decisions…associated with their values at least, recognising that it’s not always one person’s decision….”* (ENG-014, geneticist).

Relative to the French interviewees, the English interviewees focused less on the importance of formally obtaining consent from patients and put a stronger emphasis on broader principles and the decision-making process. Overall, procedures for obtaining informed consent appear to be more heterogeneous in England, including both written and verbal processes.

### Overcoming the challenges associated with communicating technical information about NIPT

Both French and English interviewees identified challenges in communicating information about the test and its results to patients from a diverse range of backgrounds. These challenges included how to communicate technical information effectively, the need for sufficient time to ensure patient understanding, and the need for well-trained healthcare professionals.

However, English and French interviewees diverged in their focus on how to address these challenges. English interviewees placed a strong emphasis on accessible language (including simplified and translated language). This was seen as particularly important when counselling patients with learning difficulties, low literacy levels, or where there may be language barriers.

*“So here at this [hospital] we have quite a good what we call a link worker service, so we have 14 in-house interpreters with a range of different languages…We have leaflets that are available in a more simpler form for women who have learning difficulties and we can use the interpreting service for people that are deaf, so we’ve got a sign language option for that.”* (ENG-001, midwife).

English interviewees referenced a range of formats for information delivery, including physical leaflets, videos, animations, websites, and other written material. Informational material was described as standardised and/or produced by a central government body. French interviewees, however, did not extensively discuss the importance of providing patients with informational written and/or translated material, with a stronger focus on time for discussion with patients.

*“In terms of documentation and translation, we have information on the college’s website [French National College of French Gynaecologists and Obstetricians] for serum markers for trisomy 21 that exists in different languages, but we don’t have anything like this for NIPT and we are limited in terms of information and translation.”* (FR-003, obstetrician gynaecologist).

English interviewees described feelings of regret and frustration about their experiences with time pressures, and how this can impact the way information is given. This was attributed to increasing amounts of information to communicate in set periods of time, as well as issues relating to staffing and resources.

*“[The midwife] should be able to discuss and give that information…but because they’re so limited in the time, like there are new tests that are introduced, and they don’t have the time in which they have all these discussions increased. For example, there is an hour to do a booking where you take history, you discuss tests, you discuss lifestyle, like it’s just too much.”* (ENG-033, midwife).

Where the English interviewees focused more on describing their frustration with such time pressures, French interviewees focused on describing the importance of, and how they ensured, having enough time for communication. Time is necessary to explain the potential outcomes and consequences of the test, as well as allowing patients space to reflect on their decision once they receive test results.

*“I take a lot of time on the phone. I spend another half an hour, an hour on the phone with the couple to discuss what it [test results] involves and to see if they have changed their minds or not or if they have made their decision if they hadn’t yet made it and to see how we proceed. So, if there is still a need for time to reflect, then we can wait. If they need to come back for a consultation to discuss again, to go into the consequences of the pathology in more details, they can come back.”* (FR-002, geneticist).

Both English and French interviewees discussed the importance of having more training for healthcare professionals. They described how such training was necessary for professionals across a range of specialities and occupations, given the presence of NIPT in differing clinical contexts (e.g. general practice, clinical genetics, obstetrics, midwifery). The potential expansion of NIPT raises challenges and a need for training for a broader range of healthcare professionals.

### Expressing values through language and practices

Both English and French interviewees explored a range of ethical issues associated with NIPT. This includes disability critiques, eugenics, and concerns around patients experiencing a pressure to test or make certain decisions regarding the pregnancy. Here we report data pertaining to how they think these issues should and could be addressed in practice.

Interviewees recognised how these ethical issues are present both within the clinical context of the individual interaction and on a broader societal level, and how these are intertwined. Their views on these issues were connected to an emphasis on the value of “choice” and/or “consent”. They discussed how these concerns could be addressed by expressing their values, or value-neutrality, through both choice of language and approaches to counselling practice.

Broadly, English interviewees emphasised the importance of being “careful” about the language that they use. This was connected to the central role of “non-directiveness” in the encounter with patients. It is essential that language is “value-neutral” and without implicit value judgements. A screening or diagnostic result is not “bad news”, but rather “just news”. This is because the very way that information is framed and communicated can impact the patients’ understanding and decision-making process.

*“The way we communicate this news and the language we use needs to change…It’s a chance of having Downs Syndrome, it’s a statistical result, it’s a number, it’s not bad news, it’s a fact, okay? The way the news is communicated to a patient can really change their feelings about it…I do feel these results could be communicated in a more neutral way…it’s not necessarily bad news, it’s just news.”* (ENG-002, laboratory manager).

*“Yeah, it’s really important to use the right language, yes. We always use the word ‘chance’ now. I’ve managed to get rid of the ‘risk’ word in all of our communications. Sadly, some of the laboratory results and even the NIPT website still has ‘risk’ when a result comes in on it…it’s high or low ‘risk’, which I think, no, it’s not, it’s ‘chance’.”* (ENG-030, midwife).

English interviewees also discussed how societal context and the broader public controversy around NIPT influences how information is presented to patients.

*“I’m always very careful and mindful about the language that’s used around these conditions. I’m aware that it can be a bit controversial and there are lots of activists and campaign groups who are against NIPT and testing for these sorts of conditions, and I’m aware that there’s a problem sometimes with language around [NIPT].”* (ENG-008, genetic counsellor).

Rather than emphasising language, framing and word choice as a means of addressing ethical concerns, French professionals focused on training professionals in the most appropriate and sensitive way to break “bad news” to patients.

*“The announcement of bad news by telephone is a no-go.” (*FR-015, obstetrician gynaecologist).

*“And unfortunately when it (the result) is positive, yes it’s bad news that has to be communicated…”* (FR-014, midwife).

However, this does not necessarily indicate that the French interviewees had more negative views towards disability than the English interviewees. The French interviewees explicitly recognised concerns relating to eugenics, and emphasised the importance of preserving individual choice in the face of potential societal pressure.

*“I hear the outcry about the possibility of eugenics. For me, the only thing that makes eugenics non-existent is the information given to the patient and the respect of individual choice. So, I also hear the argument that, in France, in particular, the care of people with disabilities is extremely poorly done. And so, that there is a very strong societal pressure is obvious.” (*FR-007, medical biologist).

### The dynamic of communication and the role of the healthcare professional

The aspects described above show some of the similarities and differences between French and English interviewees in terms of their views or perceptions of the dynamic of patient communication. In this section we will turn our focus on instances where interviewees described their understanding of the role and function of the healthcare professional in practice.

Generally, French interviewees positioned themselves somewhat more as an expert, emphasising the importance of the professional’s role in information provision including mitigating patient anxiety.

*“They come with a lot of stress, and they sometimes confuse the risk group …So, there is a lot of re-information to be given beforehand.”* (FR-010, obstetrician gynaecologist).

*“We have a colleague who follows children with Down’s syndrome and so, if families need more information before making their decision, we refer them to these colleagues so that they can discuss in greater depth what Down’s syndrome means these days, what the medical consequences are, what the intellectual consequences are, what integration to work life may look like, etc.”* (FR-002, geneticist).

Rather than focusing on consent as a product of a dynamic process, French interviewees spoke more of the importance of providing “information” as a necessary requirement for women to make their own choices.

*“I try to make sure that they [women] have this initial information to tell themselves that they are the captain of the ship. So, I’m the one giving the information and that afterwards they really have the final decision, it’s up to them. I talk to them fairly quickly about parental authority, telling them that here they are exercising, even if it’s a first child.” (*FR-013, midwife*)*.

Compared to English interviewees, French interviewees did not particularly address information that patients had received from elsewhere or emphasise the role healthcare professionals play in helping patients navigate through pre-existing information. Rather, they focused on the professional’s duty to adequately provide and explain information to patients and provide time and space for them to reflect and make their own decision.

*“I think it’s good that, in France in particular, safeguards are put in place, that we are very careful to ensure that [NIPT] is carried out correctly, with information given upfront and that the couple has time to reflect and the possibility to choose.” (*FR-002, geneticist).

English interviewees more directly emphasised the role they perceived healthcare professionals play in decision-making as a dynamic and collaborative process. They had a range of views regarding the level and quality of information that patients had access to or were aware of but also noted the changing informational landscape, as NIPT is now also offered through the NHS screening programme. Multiple interviewees indicated that they perceived a reasonably high level of general awareness among women about the existence of NIPT as an option, whether through the private sector or the NHS.

*“I think [introduction of NIPT into the NHS] was driven both by science, but also by women – public demand – because it was introduced into the private sector in the UK around 2012 and, within three years, the market absolutely boomed, and it started to be something that was spoken about on parenting forums, like Mumsnet etc. There were questions put in the UK Parliament by MPs…”* (ENG-012, community or patient representative).

English interviewees reported that some patients may come with prior awareness of the test or had sought out further information from other sources, including news outlets, social media, websites, and personal connections. This then requires healthcare professionals to navigate and assess the quality and relevance of the information that patients already had.

*“I think there’s a danger that people could think it gives much more information than it actually does…and so people might misunderstand it, but I think this is part of the education that has to go with the use of NIPT, both for the public and for the mothers and their partners, and for healthcare practitioners about what is being offered and what the positive predictive value of the test means.”* (ENG-015, geneticist).

Following from this, English interviewees reported that their perception of the role of healthcare professionals in the clinical setting had evolved from being "information-givers" into something more resembling a facilitatory or supportive role in the decision-making process; the clinical interaction is a dialogue and not just giving the patient facts.

*“So, I think nowadays our role as information-givers is much less than it used to be before the internet. In the past people came in and said, tell me about this condition, what does it mean? Nowadays they come in and say all right, I’ve watched these YouTube videos, I’ve been on that website, I’ve been on social media, and now I need help deciding.”* (ENG-013, genetic counsellor).

*“I don’t mean just giving someone a bit of information. I’ve noticed that doctors in particular will say, ‘I counselled somebody’ and what they mean is they’ve given them some facts. That’s not the same as genetic counselling. So, I think that making sure someone is given the facts but also given that emotional, that psychosocial support as well.”* (ENG-019, genetic counsellor).

## Discussion

The findings presented here provide further insight into how English and French professionals offer NIPT in the clinical setting and provide support in the decision-making process before and after testing. Within each respective sample, there were also a diverse range of views and attitudes expressed by interviewees regarding how to best support patient choice and reproductive autonomy. However, by exploring and critically comparing these perspectives, we were able to identify broader areas of congruity between English and French interviewees in terms of their key values, but also more closely examine the differing ways in which such values might be understood and translated into clinical practice.

### Similar concepts, differing lenses: choice and consent as “procedure” or “principle”

A key finding is that both English and French interviewees emphasised that the provision of NIPT must be centred on a constellation of interrelated concepts, principles and values, which included choice, freedom, autonomy, and consent. However, the way they discussed these concepts and communicated their understanding somewhat diverged. While it is important not to draw artificial delineations, these general differences were then further underscored by the ways in which English and French interviewees explained their approaches to information provision; language and practices in the patient-professional interaction; and the particular role of the healthcare professional.

Broadly, French interviewees described the practice of providing NIPT with a stronger emphasis on what we interpret here as a “procedure” lens. They emphasised the role of healthcare professionals in ensuring that certain procedures are followed and duties discharged. Relative to English interviewees, French interviewees more frequently described literally directly informing the patient that they have a choice; ensuring they understand that it is “not mandatory”; and then obtaining formal consent (which requires patients to be sufficiently/adequately informed). This “procedure” lens frames choice and consent as involving more clearly-defined or delineated aspects and tasks.

The focus on a rather procedural or formalised understanding of information and consent must be situated within the context of French legislation, and more precisely the law on patients’ rights of 2002 reforming the French medical landscape. For the first time, healthcare professionals were legally required to inform patients about their diagnosis and treatment options, and provide access to their medical records [16]. Furthermore, the 2002 law established the right for patients to make decisions about their own treatment and refuse a medical treatment or investigation, which was considered a “Copernican revolution” and represented a shift away from a paternalistic and asymmetrical doctor-patient relationship [17]. This led to a rather legalistic focus on informed consent and on presenting a treatment/investigation as “not mandatory”, also enshrined in current legislation and official documents regarding prenatal screening and diagnosis. This includes Article L2131 of the Public Health Code, as well as 2017 recommendations for trisomy 21 screening by the French National Authority for Health (Haute Autorité de santé, or HAS) [18, 19].

English interviewees more generally viewed the provision of NIPT through what we will describe here, for comparative purposes, as a “principle” lens. Rather than specific procedural aspects, English interviewees focused on ensuring choice through the application of broader principles of communication. In comparison to French legislation and policy, the English context puts less emphasis on formal criteria or approaches to obtaining consent and a stronger focus on the decision-making process of patient choice. This aligns broadly with the General Medical Council (GMC) guidance on consent and decision-making [20]. The guidance overview for the Fetal Anomaly Screening Programme (FASP) also more extensively discusses “personal informed choice” rather than consent [21].

The principle of “non-directiveness” was frequently referenced by English interviewees as being central to good practice around prenatal screening and NIPT. In the 2016 Public Health England update regarding the introduction of NIPT into the NHS, “non-directive support” was described as “very important” [22]. Non-directiveness can be understood more broadly than refraining from explicitly expressing a view or recommending a particular course of action; it can also include general framing of advice and how information is presented and communicated, both verbally and non-verbally [23]. In the prenatal context, there is also a particular focus on non-directiveness relating to patient decision-making around termination of pregnancy and promoting reproductive autonomy [24, 25]. In early years of prenatal genetics, non-directiveness was seen as critical to differentiate care from historical practices of eugenics; it has evolved over time to more nuanced conceptions that reflect the complexities of current practice [26]. However, non-directiveness has been subject to the critique that it erroneously presumes that a certain level of “neutrality” is possible in the clinical interaction [27]. There is an increasing focus on other approaches to counselling, such as shared decision-making, which integrate the healthcare professional into the process in a more collaborative role [28, 29].

In this discussion it is important to make some distinction between concepts such as choice, consent, and autonomy; although interrelated, they are different. A full examination of these is outside the scope of this paper, but is an important element in our interpretation of these data. The idea of (informed) “consent” is a concept that is generally more specifically defined, often legally, and presupposes a range of factors such as competency and voluntariness [30]. In the clinical context, it is understood as an *authorisation* by the patient for the clinician or healthcare professional to proceed with a certain treatment, intervention, or course of action [31]. However, it can be integrated into clinical practice in a range of different ways [30]. Obtaining consent (as authorisation) may be done in a less procedural way, which is reflected in the lack of emphasis English interviewees placed on specific aspects such as signing a form. However, obtaining consent can also be channelled through institutional and policy rules and processes, which is a view that is more reflective of the French interviewees’ descriptions of the process and the French legislation [18, 30].

This formalisation of the process of obtaining informed consent has been critiqued as transforming it into the performance of a “clinical ritual” [32]. Furthermore, informed consent may be *necessary* but not *sufficient* to support individual autonomy and informed choice, a view which may align with the general position of the English interviewees [32]. “Choice” as a concept is much broader, ill-defined and disputed; there are a range of views in the literature as to its definition and importance (or lack thereof) in the context of healthcare. Choice and autonomy may be understood through an individualist and/or liberal lens, which is particularly noted in the English context; other conceptualisations of choice, however, emphasise more the relational and social aspects [33–35]. It is important to examine the social and individual context in which patients make choices, and the range of complex factors that play a role in the decision-making process, which includes access to material resources [36].

### The position of the healthcare professional in the communication dynamic: “information-givers” and “decision-facilitators”

The “procedure” lens seems to position the French healthcare professional more as an “information-giver”. French interviewees placed an emphasis on discharging particular types of duties to the patients (e.g. providing detailed information) and correspondingly, respecting rights of the patient such as freedom of choice (e.g. through obtaining written informed consent and time allowed for the discussion of the different stages of the screening).

In this interpretation, the dynamic of the communication can be understood as involving a more explicit acknowledgement of an epistemic differential; a stronger delineation of roles, duties and expectations; and the process of patient decision-making as occupying a more particular and specific step in the provision of NIPT. While clinical practice in reality is of course more complex, varied and multifaceted, the way French interviewees communicated their perspective of the dynamic reflects a particular framing of the process of communication. In this framing, the healthcare professional takes time to provide complex information, which the patient then uses to ask questions and make their decision (as the “captain of the ship” in the decision-making process), and the decision is then communicated back to the healthcare professional and consent is obtained from the patient. In the patient’s decision-making process, the healthcare professional *steps back* to provide space for patients to freely exercise choice.

In comparison to the role of “information-giver”, professionals in the English context may be positioned through the “principle lens” as more fulfilling the role of “decision-facilitators”. English interviewees described their understanding of the role of healthcare professionals as helping patients navigate information and facilitating patient decision-making. They framed the overall communication process and interaction as more dynamic and collaborative. As “decision-facilitator”, the healthcare professional assists and supports the patients in making a decision that is in line with the patient’s own preferences, beliefs and values. This still involves information provision, but also imagines a broader and more integrated role in the decision-making process. Where the French healthcare professional *steps back*, in this framing, the English healthcare professional *steps in* to help patients make decisions and exercise choice. This may reflect the increasing prominence of approaches such as shared decision-making in clinical care. This perception of the role of the healthcare professional also aligns with documentation such as the GMC guidance, which states that *“You must seek to explore your patient’s needs, values and priorities that influence their decision making, their concerns and preferences about the options and their expectations…”* [20]. Research with UK midwives in the early period of (private) NIPT availability also highlighted their perception of their role as facilitators of informed choice, which involved being non-directive [37].

However, English interviewees also described difficulties in being able to translate values into the reality of clinical practice, with limited time for appointments and lack of resources. Elsewhere, research with NHS frontline healthcare workers has also described the ways in which organisational structures and policies designed to maximise efficiency can push healthcare values to the periphery, and hinder translation into practice [38]. This has the potential to result in a discrepancy between recommendations, values, and actual clinical practice. Both the English and French interviewees described the importance and necessity of having enough time to adequately inform patients. They also identified a need for more training and education for the range of healthcare professionals involved in NIPT provision, and indicated that some healthcare professionals still had insufficient knowledge for adequate communication. This is particularly important given the role of an increasing range of healthcare professionals in NIPT provision across primary and secondary care. For example, in the French context, a range of healthcare professionals (general practitioners, midwifes, obstetrician-gynaecologists) have started offering at the same cost expanded NIPT (beyond the three common trisomies) within the public health system from January 2020, as an alternative to “standard NIPT” [9].

### Communication of information and use of language

English interviewees, in comparison to French interviewees, more extensively discussed the specific way that language is used in the communication, and the principles underpinning word choice. They described the range of ways in which they ensured that their language and ways of communicating were “non-judgemental” and neutral in terms of values that are implicitly embedded in choice of language. As mentioned earlier, the importance of “value-neutral” language in prenatal testing, and non-directiveness more generally, has been described in part as an attempt to counteract historical eugenic practices in the field of genetics [39]. This might include choosing words such as “probability” or “chance” over “risk” (given the word risk inherently implies the outcome may be negative), and avoiding the use of biased or negative language such as “bad news” [40, 41]. However, as one English interviewee noted, the use of such “careful” language must be considered alongside the importance of avoiding potential patient confusion. This may be of particular concern where there is already another form of language barrier. Misunderstandings and confusion during the process of NIPT provision has the potential to cause harm to patients (for example, by making choices based on incorrect information). This does not mean that professionals should revert to using language that marginalised groups such as people with disabilities have identified as stigmatising, but rather recognising it as one factor that may play a role in the communication dynamic and the simultaneous importance of clear and unambiguous language.

Comparatively, in the French context, there was a lesser emphasis on specific word choice and neutral language, as well as what appeared to be more frequent usage of language such as “bad news” by healthcare professionals in the antenatal setting. This may be reflective of broader social and cultural differences. It has been argued that in the French context, compared to Anglophone cultures, there is historically less of a focus on the role of semantics and prescriptive language in addressing social and ethical issues [42, 43]. Furthermore, within a screening programme that has a stronger focus on test performance and improving the detection rate of T21 in order to provide the most reliable information, the identification of a fetal anomaly may be more likely to be seen as “negative” news to be told to women/couples [19].

In England, this focus on language and specific word choice is not limited to the prenatal setting within the NHS. Many of the views expressed by interviewees are not necessarily unique to the context of prenatal screening or NIPT, and must be situated within an underlying framework of values and approaches that underpins the healthcare system they operate within. For example, the NHS has a very detailed “style guide” and guidance on specific words to be chosen with regards to informational material and service delivery [44]. In the NHS, there is a strong approach of centralised standardisation of language and visual presentation, which is guided by principles such as accessibility, inclusion, and comprehension.

The English interviewees more frequently emphasised the use of such standardised informational material such as leaflets, websites, and videos in patient communication. They explained the importance of the material being translated into many languages and accessible for people with disabilities and low levels of literacy. Relative to the English interviewees, French interviewees did not emphasise the production or use of informational material in the provision of NIPT and seemed to rely more on a conversational/oral approach that involves taking time to discuss the different stages of screening in detail. In France, HAS produced a 4-page informational leaflet for women in 2018 [45]. However, it has not been translated and the distribution of the leaflet does not appear to be consistent or centralised. Broadly, the English context appears to involve a more standardised approach to information provision; this can be beneficial in reducing inequities in care and ensuring a clear standard of care, but the possibility of an over-reliance on this type of information may be less useful in complex cases. In contrast, the French context emphasises a more individualised approach to information provision. This may be beneficial in ensuring that patients receive the information that is most relevant to their individual situation and choices, but may create disparities in care where healthcare professionals receive differing levels of education and training.

English interviewees described instances in which patients indicated that they had already heard of or had pre-existing knowledge about NIPT, through avenues such as personal networks or social media. The COVID-19 pandemic may also have led to an increased use of social media for information about prenatal care by pregnant women [46]. Access to online resources and social media has led to the increasing emergence of the “expert patient”, where lay-knowledge can be reconstructed as a form of expertise [47]. This can have a range of impacts in the clinical setting. For patients, it can emphasise the importance of their experiences and reduce the power difference between patient and doctor [48]. However, the focus on informational material may also reflect an increasing expectation that patients should assume a level of individual and personal responsibility for informing themselves about their own health, although this may be problematic for people with low levels of (health) literacy [49, 50]. At a systemic level where efficiency and cost-effectiveness may be prioritised, the availability of such informational material could lead to the perception that healthcare professionals can “outsource” that aspect of communication. This may tie in to our interpretation of interviewees’ perception of the role of the English healthcare professional as being that of a “decision-facilitator”, as opposed to “information-giver”.

### Limitations and other considerations

There are some important factors to consider when interpreting or applying these results. It is necessary to recognise that these findings reflect the interviewees’ own perceptions and understandings of NIPT provision, as well as how they communicate them. These perceptions may not necessarily align with actual clinical practice, nor the perceptions of patients or other groups in society. Further research to explore the communication and information process in the context of NIPT would be useful to build on the findings presented here. Research involving more direct observations, such as an ethnographic approach, would be particularly valuable. There are also a range of recognised factors that shape multilingual qualitative research such as this study, including decisions made in the translation process [51].

## Conclusion

The introduction of NIPT into the English and French public healthcare systems has raised a range of ethical and policy issues. Many ethical concerns (such as concerns around eugenics, routinisation, and equality of healthcare access) are present in both settings. Communication and information provision in the context of NIPT and prenatal screening has been highlighted as a key area of importance, given the strong focus in both policy and the ethical literature on reproductive autonomy and informed consent [52]. However, it has been argued that a gap exists between the theoretical acknowledgment of reproductive autonomy and the way it is implemented in practice [10]. Addressing this requires a critical reflection on appropriate social policy. When formulating policies, cultural contexts that shape understandings of values and principles need to be taken into account.

One of the key observations that can be gained from the data in this study is that professionals in different systems and contexts may emphasise what appear to be, on the surface, similar values and principles in the provision of information and counselling regarding NIPT. In both the English and French contexts, supporting patient choice and decision-making was considered of paramount importance in prenatal testing. However, when these concepts are more closely interrogated, the underlying frameworks of understanding and the translation of these values into clinical practice differs. In the English context, there is an emphasis on broader principles such as non-directiveness, making efforts to standardise provision of care to ensure equity, and taking a more involved/interactive role in supporting patient decision-making based on discussion and mutual exchange. The perception of the role of the clinician is more as a “decision-facilitator”. In the French context, there is a more procedural view of communication, but also a more individualised approach. Here, the clinician is perceived as an “information-giver”, with a focus on the importance of high-quality and sufficient information to ensure that a patient provides free and informed consent.

Our research also identified areas of commonality between the French and English contexts, which can illustrate particular barriers or problems that arise in similar ways across contexts. The key concern described by both English and French interviewees was the availability of resources. Additional resources are required in terms of time, professional training, adequate staffing, and the availability of high-quality and accessible informational material for both healthcare professionals and patients. This is not a novel finding in and of itself. However, the fact that it emerged in both contexts illustrates that without appropriate and sufficient resources, regardless of the approach to communication, it is difficult to provide high quality care. Both French and English interviewees emphasised the importance of having enough time to fully inform patients, and described the difficulties of translating their values into clinical practice in a context where pressure on resources is only increasing. It is not sufficient for governments, healthcare bodies, and professional organisations to produce precisely worded guidelines and carefully thought-out policies regarding the provision of NIPT. It is also necessary for healthcare professionals to have the time, training or resources to apply these guidelines and policies to clinical practice.

Through our comparative qualitative research, we have generated findings that may have a range of useful implications and benefits for the offer of NIPT in the clinical setting across different social and cultural contexts. Our key finding is that even where professionals report broadly similar values across different cultures, the perception, understanding and translation of these values into practice can differ in significant and context-dependent ways. Our work here can serve both as a basis for future research as well as informing policy and clinical practice relating to NIPT. By comparing English and French professionals’ views, experiences and values, this research can also illustrate possible advantages and disadvantages of different approaches to support decision-making and reproductive autonomy in the provision of NIPT. This knowledge can inform broader policies of prenatal genetic testing and be beneficially translated into other settings.

### Electronic supplementary material

Below is the link to the electronic supplementary material.

Supplementary Material 1

Supplementary Material 2

## Data Availability

Data are available from the UK Data Archive for researchers who meet the criteria for access to confidential data: Horn, Ruth (2023). Non-invasive Prenatal Testing Study: Comparison England, France, Germany, 2021–2022. [Data Collection]. Colchester, Essex: UK Data Service. 10.5255/UKDA-SN-856508. https://reshare.ukdataservice.ac.uk/856508/.
