# Supplementary material for: Supporting patient decision-making in non-invasive prenatal testing: a comparative study of professional values and practices in England and France
Source: BMC Med Ethics. 2024 Mar 21;25:34. doi: 10.1186/s12910-024-01032-0 (PMC10956335; doi:10.1186/s12910-024-01032-0)
Supplement: Supplementary file 2 — Supplementary Material 2 [file 12910_2024_1032_MOESM2_ESM.docx]

**Topic guide 2: semi-structured interviews with policy-makers**

The questions will be addressed using semi-structured interviews. This means that the questions will be open to allow the person's point of view to emerge and to be taken in consideration. Through the interviews, we will collect participants’ views, experiences and values in their way of expressing themselves, making criticisms, judging what seems important to them, what are expectations, and what is a cause of concern. During the interview, questions may be formulated as follows, with the aim of supporting the participant's subjectivity and not imposing ways of thinking about NIPT.

This is not intended to be a precise script. The following questions are illustrative of the kind of questions which will be asked at interview.

**QUESTIONS:**

**Prenatal testing and introduction of NIPT into routine examinations**

1. (If someone is/was involved at policy-level) We had contacted you because…. (possible role). Could you tell us a bit more, what is/was your role here?
2. (If someone was involved in a policy decision/report) How do you feel about this decision/report, could you say a little more about it?
3. What do you think of NIPT?
4. What do you think are the benefits of NIPT?
5. In your opinion, what are the difficult issues related to NIPT, particularly with regard to its introduction into routine prenatal care? Do you see any questions or problems here that are particularly relevant from an ethical point of view?
6. What impact do you think the introduction of NIPT will have on routine prenatal care?
7. Do you think NIPT is significantly different from other screening tests?
8. (If someone raised ethical/other issues) How do you think the issues you raised in relation to NIPT could be addressed and what would need to change?

**Information and discussion**

1. How do you think NIPT should be offered? What do you think is important to communicate to women when NIPT is offered?

**Communication of results and decisions**

1. Do you think there is anything that needs to be changed to support women make reproductive decisions based on the test? And what?

**Future developments of NIPT**

1. Do you think NIPT should also be available for other genetic abnormalities apart from those already performed? If so, which ones and why?
2. How do you see the future development of NIPT? Do you think there is scope for wider use in genomic medicine, and how?
3. Are there any other topics that we have not covered that you would like to discuss and that you feel are relevant to NIPT and the development of best practice models?

**The researchers pay particular attention to topics such as:**

- Counselling and support given
- Decision-making
- Consent
- Information
- Autonomy of women and couples
- Role of professionals
- Practices associated (or not) with laws, regulations and guidelines
- Advantages/risks associated with NIPT/the way it is implemented
- Value of the test
- Future development of the NIPT and its use in genomic medicine
- Other ethical issues
